# Intrapericardial recombinant tissue plasminogen activator in purulent pericarditis- case series

**DOI:** 10.1186/s12872-020-01674-z

**Published:** 2020-08-27

**Authors:** Małgorzata Dybowska, Monika Szturmowicz, Lucyna Opoka, Piotr Rudziński, Witold Tomkowski

**Affiliations:** 1 I Department of Lung Diseases, National Tuberculosis and Lung Diseases Research Institute, ul Plocka 26, 01-138 Warsaw, Poland; 2 Department of Radiology, National Tuberculosis and Lung Diseases Research Institute, Plocka 26, 01-138 Warsaw, Poland; 3 Department of Thoracic Surgery, National Tuberculosis and Lung Diseases Research Institute, Plocka 26, 01-138 Warsaw, Poland

**Keywords:** Purulent pericarditis, Constrictive pericarditis, Subxiphoid pericardiotomy, Fibrinolysis

## Abstract

**Background:**

Pericardial constriction is one of the complications of purulent pericarditis (PP). Most difficult to treat, which may develop both in early and in the late period of the disease, resulting in a very poor prognosis.

**Case presentation:**

We present case series of 4 patients with purulent pericarditis, in whom direct intrapericardial administration of recombinant tissue plasminogen activator (r-tPA) was used.

Management of PP requires a combined surgical and medical approach. The most important is complete drainage of the effusion by subxiphoid pericardiotomy connected with complementary use of broad-spectrum antibiotics. Despite the use of broad- spectrum antibiotics, in some patients a large volume of daily drainage is still present. Constrictive pericarditis as a complication of PP is observed in majority of patients.

Intrapericardial administration of fibrinolytic agents, although not strongly recommended, can improve efficacy of antibiotic treatment especially in patients with loculation fluid and can prevent the development of constrictive pericarditis.

r-tPA was applied at a dose of 20 mg dissolved in 100 ml of normal saline in a 100 ml syringe, administered by a large pericardial drain (Pezzer drain) installed into the pericardial cavity during pericardioscopy. The tube was closed and re-opened after 24 h.

No serious complications, such as bleeding, allergy or hypotension, were noted.

**Conclusion:**

We present case series of 4 patients with purulent pericarditis, in whom direct intrapericardial administration of recombinant tissue plasminogen activator (r-tPA), prevented the development of constrictive pericarditis, and increased efficacy of antibiotic treatment without any significant complications.

## Introduction

Purulent pericarditis (PP) is a rare cause of pericardial disease, with the prevalence of less than 3%, but with potentially life-threatening complication [1]. Most frequently PP develops in the course of bacterial infections of the head, neck or respiratory tract, especially in immunocompromised hosts [2–4]. The other risk factors are chest surgery or injuries [2–4].

The recommended treatment of PP is based on pericardial drainage and intravenous antibiotics administration [3].

Intrapericardial use of fibrinolytic agents such as Streptokinase (SK) or Urokinase (UK) is suggested in case of early signs of pericardial constriction, fibrin deposits and loculation of the fluid [3].

Despite proper multidirectional treatment, PP still has a 20–30% mortality rate [2]. In the early stage of the disease, mortality is caused by cardiac tamponade and/or septic shock [3–6]. Late mortality in the course of PP is combined with pericardial constriction [2]. Constrictive pericarditis is characterized by loss of elasticity of the pericardium due to progressive fibrosis and in some cases - calcification. Reduced flexibility of the pericardium causes the increase of heart filling pressures and finally heart failure. Pericardiectomy, which is the treatment of choice in the case of constrictive pericarditis, exposes the patient to the possibility of complications and is combined with a mortality rate of about 4,9–8% [7, 8].

Clinical observations based on a small group of patients suggest that the intrapericardial administration of fibrinolytic drugs, although not strongly recommended, is an effective way to prevent the development of pericardial constriction and thus protects the patient from a pericardiectomy [2, 3]. Up to now, no randomized studies, confirming the efficacy of such treatment, have been performed.

We present 4 patients with purulent pericarditis, successfully treated with intrapericardial administration of recombinant tissue plasminogen activator.

## Case presentation

The clinical data of 4 patients with confirmed PP, who were treated with intrapericardial recombinant tissue plasminogen activator (r-tPA) is presented in Table 1. The group of patients consists of 3 males and 1 female, age 27 to 69 years.

**Table 1:** Clinical data of 4 patients with recognised purulent pericarditis

	Case 1	Case 2	Case 3	Case 4
Sex	male	female	male	male
Age	27	50	69	63
Predisposingfactors	common variable immunodeficiency left-sided pneumonia empyema sepsis CMV infection	not identified	not identified	bacterial inflammation of the knee-joint
Responsible pathogen	*Streptococcus pneumoniae*	*Staphylococcus epidermidis*	not identified	*Staphylococcus aureus*
Indications for intrapericardial fibrinolytic treatment	echocardiographic features of early constriction	a large amount of fibrin in the pericardium with fluid loculations	echocardiographic features of early constriction	large pericardial drainage
Type of intrapericardial treatment	r-tPA (Actylise) 20 mg/100 ml 0,9% NaCl clamped tube for 24 h	r-tPA (Actylise) 20 mg/ 100 ml 0,9% NaCl clamped tube for 24 h	r-tPA (Actylise) 20 mg **/**100 ml 0,9%NaCl clamped tube for 24 h	17 days after Streptokinase administration (ineffective)
				r-tPA (Actylise) 20 mg/100 ml 0,9% NaCl clamped tube for 24 h
Number of r-tPA doses	1	2	1	2
Indications for repeated intrapericardial fibrinolytic treatment	–	a large amount of fibrin in the pericardium with fluid loculations	–	large pericardial drainage
				echocardiographic features of early constriction
Type of repeated intrapericardial treatment	–	r-tPA (Actylise) 20 mg/ 100 ml 0,9% NaCl clamped tube for 24 h	–	r-tPA (Actylise) 20 mg/ 50 ml 0,9% NaCl clamped tube for 24 h
Local complications of intrapericardial fibrinolytic treatment	–	extensive leak of pericardial fluid next to the drain after first r-tPA dose	–	–
General complications	not observed	not observed	not observed	not observed
Inflammatory biomarkers				
CRP mg/l	269	27	231	292
WBC [x10̂^9^/l	36	20	13,5	15
Procalcitonin ng/ml	1,75	no data	no data	no data
Intravenous antibiotics doses/days				
	Meropenem/3 × 1,0 g/15 days	Ceftriaxon/1 × 2,0 g/5 days	Piperacillin/Tazobactam/3 × 4,5 g/ 28 days	Piperacillin/ Tazobactam/3 × 4,5 g/21 days
	Vancomycin3x1,0 g/13 days	Clarithromycin/2 × 500 mg/22 days	Levofloxacin/2x500mg/28 days	Ciprofloxacin/2 × 200 mg/ 8 days
	Colistin/3x2mlnIU/11 days	Doxycycline/1x100mg/35 days	Amikacin/2x500mg/28 days	Linezolid/2 × 600 mg/13 days
	Clindamycin/3 × 600 mg/8 days		Augmentin/3 × 1,2 g/ 24 days	
	Metronidazole/ 3x250mg/9 days			
	Ganciclovir/2x200mg/13 days			
Length of drainage	29 days	19 days	17 days	32 days
Time from intrapericardial r-tPA to the drain removal	8 days	6 days after the second r-tPA dose	8 days	6 days after the second r-tPA dose

PP was recognized in the patients who were suffering from generalized weakness, dyspnoea, fever, chest pain and - in one case- septic shock. Pericardial effusion (hyperechogenic fluid around the whole heart and in some cases with a large amount of fibrin) was diagnosed by echocardiography. The purulent character of the fluid was confirmed by physical and biochemical examination (turbid characteristics, low glucose level, high LDH and high WBC count with an increased number of neutrophils).

In one patient purulent pericarditis developed in the course of bacterial knee -joint inflammation, in another, diagnosed with common variable immunodeficiency, in the course of pneumonia and empyema. In the remaining two patients, the cause of pericardial infection has not been identified.

The purulent pericardial fluid was obtained from all of the patients, the cultures of the fluid tested positive for MSSA in one patient and for *S. epidermidis* in another. Because of the fact that S.epidermidis was diagnosed in two different fluid cultures, it was considered a cause of purulent pericarditis and not a contamination. In the other two patients, the cultures of pericardial fluid were negative, probably due to previous antibiotic therapy.

Subxiphoid pericardiotomy under general anesthesia has been performed in all of the patients with implantation of a large tube drain (Pezzer drain). The indications for intrapericardial fibrinolytic treatment (IFT) were: prolonged purulent drainage and fibrin deposits in pericardium (4 patients), early echocardiographic signs of pericardial constriction despite optimal treatment (3 patients), excessive pericardial drainage with signs of pericardial fluid inoculation (1 patient- case 4). Early echocardiographic signs of constriction were defined as: thickening of pericardium, the mitral inflow velocities showing a pseudo-normal or restrictive filling pattern (E/A > 1 and deceleration time < 150 ms), the respiratory variation in filling velocities, inferior vena cava plethora with expiratory hepatic vein diastolic reversal wave.

r-tPA was applied at a dose of 20 mg dissolved in 100 ml of normal saline in a 100 ml syringe, administered by a large pericardial drain (Pezzer drain). The tube was closed and re-opened after 24 h.

Two patients received a the single dose of r-tPA, and the remaining two – two doses.

In one case (case 2) it was related to an extensive leak of pericardial fluid next to the drain, and the necessity of early opening of the drain, in another one (case 4) – with the persistence of a large amount of fibrin in the pericardium with fluid loculations. The repeated intrapericardial fibrinolysis in this patients was caused by excessive pericardial drainage.

The treatment has been effective in all of the patients, diminishing the echocardiographic signs of early constriction in 3 patients, and reducing large pericardial drainage in the remaining one. The drains were removed 6–8 days after the last dose of r-tPA.

The decision to remove the drain from the pericardium was made based on the reduction of daily drainage to less than 20 ml, and echocardiographic signs of remission (fluid withdrawal and regression of early constriction features).

In each of these four cases, the successful treatment of purulent pericarditis was achieved.

**Fig. 1:**
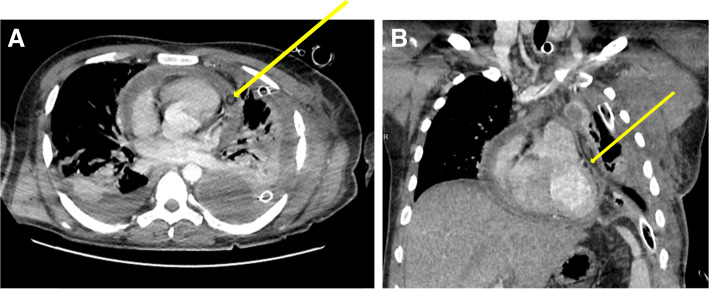
Chest CT performed before intrapericardial r-tPA treatment. Purulent pericarditis and pleuritis in the course of common variable immunodeficiency syndrome. Pericardial effusion, the layer of 17 mm. Thickening of the pericardium up to 4 mm. Drain in the pericardial sac - arrow. Bilateral pleural effusion, drain in the left pleura. Large atelectasis in the lower parts of the lungs due to pleural effusion. **a** – Mediastinal window, axial view. **b** – Mediastinal window, frontal view

**Fig. 2:**
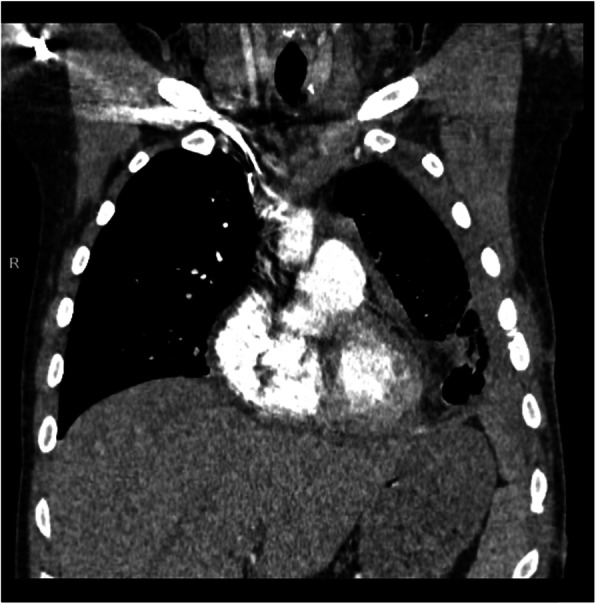
Chest CT performed 27 days after intrapericardial r-tPA treatment (mediastinal window, frontal view). Pericardial effusion, the layer of 5 mm, normal thickness of pericardium. Left sided pleural effusion with inoculations

Chest computed tomography scans before and after intrapericardial r-tPA therapy in the patient with purulent pericarditis in the course of pneumonia and empyema due to common variable immunodeficiency syndrome were presented in Figs. 1a-b and 2.

An MRI was not performed due to the lack of availability and long duration of the study -two patients were in severe condition, one was in septic shock.

Additionally MRI is not listed in 2015 guidelines as a first line diagnostic method for patients with PP.

It is a valuable procedure to confirm constrictive pericarditis for stable patients, before surgery-pericardiectomy. All cases were monitored by bedside echocardiography every day.

No serious complications, such as bleeding, allergy or hypotension, were noted. In one case, due to extensive leak of pericardial fluid next to the drain, it was reopened after 5 h.

An appropriate, broad-spectrum antibiotic therapy was used intravenously, on average – for 21 days.

In all of the patients- intrapericardial administration of fibrinolytic agents, caused remission of early sings of pericardial constriction, and regression of pericardial effusion.

The patients were observed for 10 months (case 1), 13 months (case 2), 30 months (case 3) and 7 years (case 4) respectively. The repeated echocardiographic examinations revealed no signs of pericardial constriction, at follow –ups.

## Discussion and conclusions

We presented 4 cases of purulent pericarditis, treated successfully with intrapericardial r-tPA.

Management of PP requires a combined surgical and medical approach. The most important is complete drainage of the effusion combined with complementary use of broad-spectrum antibiotics. Because of loculations and adhesions simple evacuation by surgical or percutaneous drainage may not prevent constrictive pericarditis [9]. Open surgical drainage through subxiphoid pericardiotomy is preferable [5]. Pericardiostomy decreased mortality to about 50%, and a further decrease of mortality to 30% has been associated with intravenously antibiotics use.

The guidelines of the European Society of Cardiology from both 2004 and 2015 recommend intrapericardial use of fibrinolytic agents in the therapy of PP, however the class of recommendations is low (IIa) as well as the level of scientific evidence (C) [3, 5]. Moreover, the optimal type of fibrinolytic agent, dose, method of administration, the volume of the infused solution, used in direct, pericardial treatment are still not known [3, 5].

As PP is a rare entity, the randomized clinical trials are lacking and all the information concerning treatment efficacy and safety comes from case reports or case series.

The clinical experience with IFT has been summarized by Augustin et al. in 2011 [2] and recently by Wiyeh et al. [4]. Majority of the patients have been treated with intrapericardial streptokinase (SK) [2, 4].

Long term effect has been achieved in 86–89% of patients [2, 4]. Several non-fatal complications have been noted: one case of cardiac tamponade due to bleeding, several cases of minor bleeding and occasionally – hypotension, febrile reaction, and fistula formation [2, 4]. Because of the risk of allergy and immunization, using UK or r-tPA has been suggested [10].

Our own experience with SK used as IFT, concerns 4 cases, in one of them severe intrapericardial bleeding occurred as a complication, and in one case the therapy was ineffective and the patient received r-tPA as second line treatment (case 4) [11, 12]. Major haemorrhage due to instillation of SK were also reported by other authors [2]. One case of haemorrhagic tamponade after SK fibrinolysis was described in a child [13].

In comparison to the above mentioned data from the literature [2, 4], the efficacy of intrapericardial r-tPA, reported in the present case series was non-inferior to SK. Nevertheless it is difficult to compare these data, due to a small number of patients, as well as different indications for IFT. Our indications consisted mainly of signs of early constriction, whereas other authors used IFT in case of drainage ineffectiveness with signs of cardiac tamponade in the course of PP.

The safety of intrapericardial r-tPA was high, no complications have been observed in our patients, apart from a pericardial leak in one case probably due to the large volume of purulent fluid released from loculations.

Use of r-tPA allows subsequent administration of the same drug (case 2 and case 4 were treated with repeated intrapericardial dose of r-tPA without any complications after second administration), because of no immunization.

Another important aspect concerns the timing of IFT in case of early constriction. As fibrin formation occurs during the first week of PP, fibrinolytic therapy should be used early, preferably in the first two weeks of the disease, however there in no consensus on the appropriate timing of fibrinolysis [2]. It should be pointed out that pericardiectomy is recognized as a surgical technique that provides significant improvement and survival, but is combined with high mortality [8].

Use of r-tPA significantly decreased signs of constrictive pericarditis and decreased necessity of surgical treatment in cases with PP [11].

Moreover, in the literature, there is no consensus on what dose and how intrapericardial fibrinolysis should be administered.

The limited data available which is based on individual case reports present a range of doses from 2 mg/ 10 ml or 30 mg in 1 dose [2].

Moreover, limited data concerning r-tPA dose for the treatment of empyema suggested a dose of 10 mg [14, 15].

In our schema, based on the 4 cases, we proposed 20 mg r-tPA diluted in 100 ml normal saline and clamping the drain for 24 h.

Without prejudice to the efficacy and safety of direct, intrapericardial administration of fibrinolytic agents in cases with confirmed PP, we would like to recommend, the described above schema consisting of 20 mg of r-tPA dissolved in 100 ml of saline in patients with previously performed subxiphoid pericardiostomy followed by insertion of the large drain connected with broad-spectrum antibiotics. We have included our recommendations in algorithm. Such therapy seems to be safe and effective in case of early pericardial constriction in the course of PP.

## Supplementary information

**Additional file 1.** Algorithm.

## Data Availability

Available from the authors in source materials and clinical data at the National Tuberculosis and Lung Diseases Research Institute, Plocka 26, 01–138 Warsaw Poland.

## Abbreviations

PP: Purulent pericarditis
r-tPA: Recombinant tissue plasminogen activator
IFT: The indications for intrapericardial fibrinolytic treatment
SK: Streptokinase
UK: Urokinase
