# Supplementary material for: Intrapericardial recombinant tissue plasminogen activator in purulent pericarditis- case series
Source: BMC Cardiovasc Disord. 2020 Aug 27;20:392. doi: 10.1186/s12872-020-01674-z (PMC7451238; doi:10.1186/s12872-020-01674-z)
Supplement: Supplementary file 1 — Additional file 1. Algorithm. [file 12872_2020_1674_MOESM1_ESM.docx]

| **Suspicion of purulent pericarditis**  predisposing factors: chest surgery, chest infections, immunodeficiency  symptoms: fever, chest pain, dyspnea  signs: hypotonia, tachycardia, pulsus paradoxus  increased CRP, WBC, PCT |
| --- |

| Other diagnosis |
| --- |


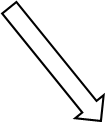


Yes

No

| Echocardiography:  large amount of hyperechogenic pericardial fluid  or pericardial fluid with  loculations and fibrin deposits  signs of cardiac tamponade |
| --- |

| urgent evacuation of fluid by pericardiotomy/pericardioscopy  and  macroscopic confirmation of purulent fliud |
| --- |

Yes

No

Other diagnosis

No

Yes

Fluid evacuation without implantation of large drain

| implantation of a large drain  (Pezzer drain)  into the pericardial space  and  administration of broad-spectrum antiobiotics intravenously  for at least 15-21 days |
| --- |

| Pericardial fluid loculation of residual effusion  or/ and  Persistent large drainage ( more than 50 ml/day )  or/and  Present fibrin deposits on visceral pericardial layer and fibrin adhesions in pericardial space  or/and  Echocardiographic signs of early pericardial constriction |
| --- |

Yes

| Instillation of fibrinolytic agent:  r-tPa 20 mg dissolved in 100 ml normal saline in a large 100ml syringe  administer by large pericardial drain ( Pezzer drain) |
| --- |

Yes

| Clamp tube drain for 24 hours |
| --- |

| Loculations, residual effusion  or/and  Persistent large drainage ( more than 50 ml/day )  or/and  Present fibrin deposition on visceral pericardial layer and fibrin adhesions in pericardial space  or/and  Echocardiographic signs of early pericardial constriction |
| --- |

No

Yes

Remove drain

| Consider second instillation of fibrinolytic drug  r-tPa 20 mg dissolved in 100 ml or in 50 ml normal saline in large 100ml syringe  administer by large pericardial drain ( Pezzer drain) |
| --- |

| Clamp tube drain for 24 hours |
| --- |

| Remove the drain when drainage decreased to 20 ml/day or less |
| --- |

| Echocardiographic signs of pericardial constriction? |
| --- |

No

Yes

Repeat echocardiography every 3 months

MRI signs of pericardial constriction?

Yes

| Consider surgery: pericardiectomy |
| --- |
